# Placental implantation over prior cesarean scar causes activation of fetal regulatory T cells

**DOI:** 10.1002/iid3.214

**Published:** 2018-02-12

**Authors:** Tina A. Nguyen, Daniel A. Kahn, Andrea I. Loewendorf

**Affiliations:** ^1^ Division of Maternal‐Fetal Medicine, Department of Obstetrics and Gynecology David Geffen School of Medicine,UCLA Los Angeles California; ^2^ Asante Physician Partners Medford Oregon; ^3^ Huntington Medical Research Institutes Reproductive − and Vascular Immunology Pasadena California

**Keywords:** Cesarean section, chimerism, non‐inherited maternal antigens (NIMA), placenta, regulatory, T‐lymphocytes

## Abstract

**Introduction:**

Maternal‐fetal chimerism is miniscule, a testament to the integrity of the uteroplacental interface. The soundness of this border region is potentially altered through cesarean delivery of prior babies with uncertain consequences for the following pregnancies.

**Methods:**

Using multicolor flow cytometry and quantitative PCR of non‐inherited maternal antigens we performed a retrospective case control pilot study and formulated the null hypothesis that placental implantation over a prior uterine scar does not result in the presence of memory Treg (CD45RO+) in the fetus. We then performed a power calculation and performed a blinded, appropriately powered prospective case control study to test the null hypothesis.

**Results:**

Fetuses born to mothers with prior uterine scar have a roughly five times higher maternal to fetal microchimerism when the placenta directly interacts with the uterine scar. Unlike exposure to antigens in adult life, in utero antigenic exposure induces tolerogenic (Treg) responses in fetuses and we here report the presence of fetal Treg with a memory phenotype (CD45RO+). However, we only find such CD45RO+ fetal Tregs when the placenta abuts the uterine scar (Risk Ratio = 5 [*p* < 0.05 CI:(1.448 to 17.27)]). These memory fetal Tregs are functionally highly suppressive compared to CD45RA‐expressing fetal Tregs, and have specificity for non‐inherited maternal antigens.

**Conclusions:**

We found that uterine scars, in the case of our study these scars are from prior c‐sections, fundamentally impair uterine integrity allowing for increased antigen exposure of the fetus; with our appropriately powered study we rejected the null hypothesis and accepted the alternative hypothesis that placental implantation over a prior uterine scar results in the presence of memory Treg (CD45RO+) in the fetus. Thus, our study demonstrates a previously unappreciated role for uterine integrity in limiting fetal antigenic exposure, a key element to avoid the formation of inappropriate tolerances by the fundamentally tolerogenic fetal immune system.

## Introduction

How humans are born is changing. Historically, before the advent of safe cesarean delivery, women with contraindications for vaginal birth suffered high rates of morbidity and mortality. More recently, the circumstances that prompt or warrant cesarean delivery have been ever expanding and now include maternal request 1. In the United States, 1/3 of all births occur by cesarean section 2. However, surgical birth implies short‐ and long‐term consequences for both mother and child 3, 4. A woman with a history of a cesarean delivery has increased risks in subsequent pregnancies that are associated with a scarred uterus 4. Neonatal outcomes associated with development in a “scarred” uterine environment have been evaluated only in the context of the maternal risks of uterine scar rupture or abnormal placentation (e.g., accreta) 4.

The human fetal immune system develops the ability to mount adaptive responses by mid‐gestation 5. The uteroplacental interface is an incomplete barrier and trafficking of cells between both compartments is easily detectable 6. Since mother and fetus are antigenically disparate individuals (in the vast majority of human pregnancies), the development of immune tolerance is essential 7, 8. To cope with the potential to mount an inflammatory response against non‐inherited maternal antigens (NIMA), fetal adaptive responses are dominated by the generation of T regulatory (Treg) responses by an uncertain mechanism(s) 9. These Tregs with specificity for NIMA are long‐lasting and have been shown to be functionally suppressive well into adulthood 9. Thus, in utero fetal immune responses shape the lifetime functional immune profile of an individual.

In this study, we investigate the possibility that a uterine scar may impact the developing fetal immune responses. When the placenta implants in the region of a uterine scar, the potential for a greater degree of invasion that can cause life‐threatening hemorrhage at delivery (e.g., placenta accreta) increases 10. Since the placenta‐uterus interaction may be substantively altered by a scar, we hypothesized that such interactions may alter the degree of maternal‐fetal microchimerism and be reflected in changes to the fetal immune repertoire.

## Materials and Methods

### Human subjects

Healthy women scheduled for repeat cesarean section in the third trimester with singleton pregnancies were recruited for participation between March 2009 and July 2014. Demographic and obstetrical characteristics from the recruited population are provided in Tables 1 and 2.

**Table 1:** Subject demographics and obstetrical characteristics in the clinical study (Fig. 2D)

	Anterior (*N* = 10)	Posterior (*N* = 10)	*p* value
Age	34.9	34.3	0.769
Gravity	4	3.2	0.655
Parity	1.4	1.3	0.774
Ethnicity			
Caucasian	5	6	
Asian	0	2	
Black	1	0	
Hispanic	4	2	
Significant Comorbidites			
AMA			
GDMA1			
Mode of Delivery			
Repeat C/S	6	5	
VBAC	4	5	

*N* = 20.

Risk difference 80%­.

Risk ratio 5 [*p* < 0.05 CI:(1.448 to 17.27)].

Age, gravidity, parity, fetal birth weight, and APGAR score differences were analyzed with a two‐way Student's *T* test. Ethnicity, advanced maternal age differences and mode of delivery were analyzed using a Fisher's exact test.

**Table 2:** Subject demographics and obstetrical characteristics (Figs. 1A; 2B and C; 3A, B)

	Anterior (*N* = 10)	Posterior (*N* = 10)	*p* value
Age	34.9	34.3	0.769
Gravity	4	3.2	0.655
Parity	1.4	1.3	0.774
Ethnicity			
Caucasian	5	6	
Asian	0	2	
Black	1	0	
Hispanic	4	2	
Significant Comorbidities			
AMA			
GDMA1			
Mode of Delivery			
Repeat C/S	6	5	
VBAC	4	5	

Age, gravidity, parity, fetal birth weight, and APGAR score differences were analyzed with a two‐way Student's *T* test. Ethnicity, advanced maternal age differences and mode of delivery were analyzed using a Fisher's exact test.

### Tissue collection

Cord blood was collected after delivery of the fetus, but prior to placental separation, into sterile cord blood collection kits containing Citrate Phosphate Dextrose solution (Medsep Corporation, Covina, CA).

### Lymphocyte purification

Granulocytes were depleted utilizing the RosetteSep Granulocyte Depletion Cocktail (Stemcell Technologies, Vancouver, Canada) following manufacturer's recommendations. Granulocyte‐depleted mononuclear cells were isolated by gradient centrifugation over Ficoll‐Paque PLUS from GE Healthcare (Uppsala, Sweden) following manufacturer's recommendations. Cells were washed twice with sterile PBS and enumerated utilizing an Accuri flow cytometer with Propidium Iodide exclusion of dead cells.

### In vitro suppression assay

**Figure 1:**
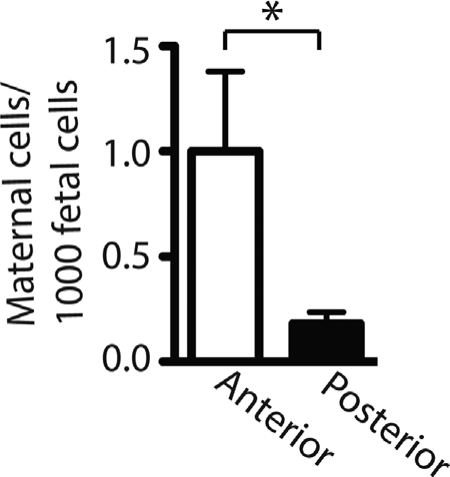
Fetuses with anterior placentas implanted over prior cesarean section scars have increased maternal microchimerism. Fresh granulocyte reduced cord blood samples from fetuses born to women with prior cesarean sections. Maternal microchimerism as determined by pPCR. Student's *T* test, unpaired. *p = 0.029.

Freshly isolated, granulocyte‐reduced cord blood T lymphocytes were isolated using the EasySep™ Human T Cell Enrichment Kit (Stemcell Technologies) and stained with the antibody panel depicted in Table 3. CD3+CD4+CD8‐CD25hiCD45RAhi and CD3+CD4+CD8‐CD25hiCD45ROhi cord blood Treg were sorted with a Becton Dickinson FACSAriaIII or Becton Dickinson FACSAriaII. 1 × 10e5 untouched cord blood cells were seeded as responders with the stimulator Conconavalin A (5 μg/ml, Fig. 3A) or 1 × 10e5 maternal cells exposed to 5000 Rads in a Cs^132^ irradiator (Fig. 3B). No cells, 1 × 10e4 CD45RAhi or 1 × 10e4 CD45ROhi Treg were added and cells were cultured in RPMI 1640 supplemented with 10% (vol/vol) heat‐inactivated FBS, 5.5 × 10^−5^ M β‐mercaptoethanol, 100 U/ml penicillin, 100 U/ml streptomycin, and 2.5 mM L‐glutamine. Cells were cultured at 37°C with 5% CO_2_ in a humidified incubator for 72 or 96 h. 1 μCi of ^3^[H] Thymidine was included for the final 18–22 h of the culture. The co‐culture setup is detailed in Table 4.

**Table 3:** Antibodies and instrument configuration in cell sorting

Antigen	Source	Clone	ng/test	Fluorochrome	Excitation (nm)	Longpass Dichroic Mirror	Bandpass filter
CD4	Biolegend	OKT4	180	Brilliant violet 510	407	blank	450/40
CD45RA	eBioscience	HI100	400	AF488	488	502LP	530/30
CD8a	eBioscience	SK1	125	PerCPeF710	488	690LP	675/20
CD25	eBioscience	BC96	125	PE	633	556LP	585/42
CD3	eBioscience	OKT3	500	AF700	633	685LP	710/50
CD45RO	eBioscience	UCHL1	600	APC	633	blank	670/20

**Table 4:** Setup of the in vitro suppression assay

Figure	Depiction	Responders	Stimulation	Treg addition
3A	Black bar	1 × 10e5 total fetal cells	ConA	No
3A	White bar	1 × 10e5 total fetal cells	ConA	1 × 10e4 CD45RAhi fetal Treg
3A	Gray bar	1 × 10e5 total fetal cells	ConA	1 × 10e4 CD45ROhi fetal Treg
3B	Black bar	1 × 10e5 total fetal cells	1 × 10e5 maternal lymphocytes	No
3B	White bar	1 × 10e5 total fetal cells	1 × 10e5 maternal lymphocytes	1 × 10e4 CD45RAhi fetal Treg
3B	Gray bar	1 × 10e5 total fetal cells	1 × 10e5 maternal lymphocytes	1 × 10e4 CD45ROhi fetal Treg

### Phenotypic analysis via multicolor flow cytometry

**Figure 2:**
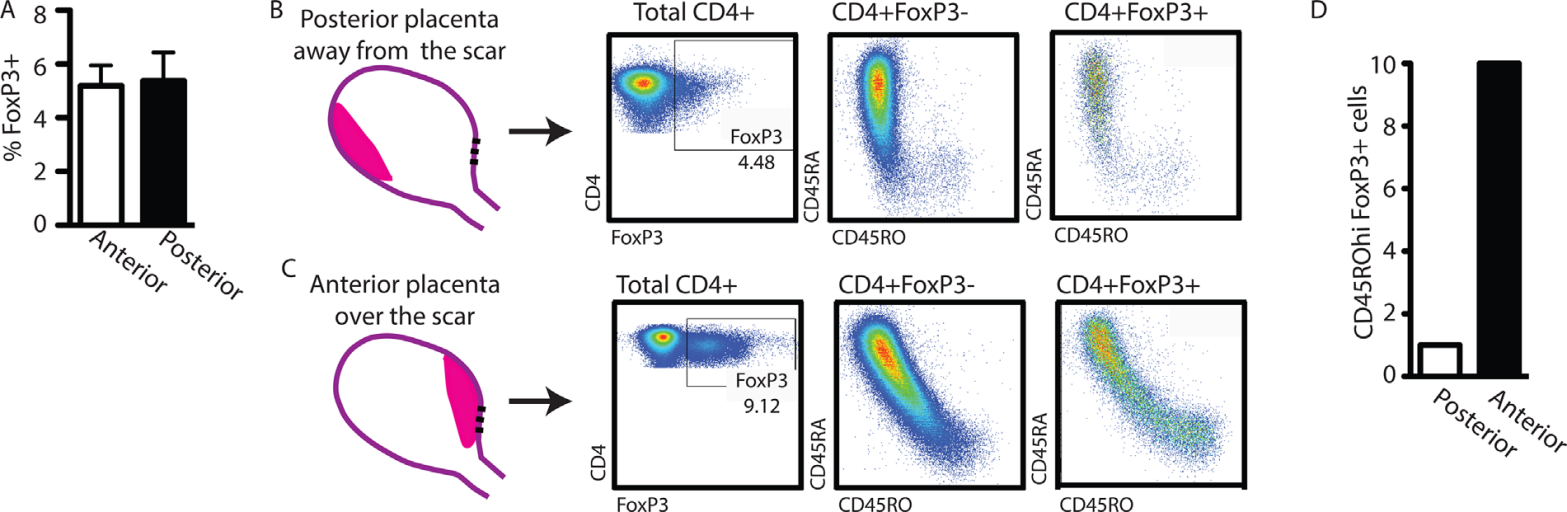
Fetuses with anterior placentas implanted over cesarean section scars have activated Treg. Fresh granulocyte‐reduced cord blood samples from fetuses born to women with previous cesarean sections. (A) Total percentage of FoxP3+ cells within the CD4+ population. (B and C) CD45RO and CD45RA expression on the surface of CD4+FoxP3‐ (middle dot blots) or CD4+FoxP3+ (right dot blots) T cells in cord blood lymphocytes from fetuses with posterior (B) or anterior placentas (C). (D) Risk difference for cord blood lymphocytes from fetuses with posterior (white bar) or anterior (black bar) placental implantation locales. (A) Student's T test, unpaired *p* = 0.8879. (D) Risk Ratio = 5 [*p* < 0.05 CI:(1.448 to 17.27)].

Phenotypic characterization as in Figure 1B and C and Supplemental Figure S1 was carried out as previously published utilizing the antibodies and concentrations listed in the Table 5 11. Analysis was performed on a BD SORP LSR II analytic flow cytometer. Post‐acquisition analysis was performed with FlowJo (Treestar, Palo Alto, CA). Flow cytometric characterization for the clinical study (Fig. 2D, *n* = 20) was performed on a BD Accuri C6 flow cytometer (BD Biosciences, San Jose, CA) using the antibody panel listed in Table 6. Post‐acquisition analysis was performed with BD CSampler Software (BD Biosciences).

**Table 5:** Antibodies and instrument configuration used for antigen detection for Figure 2B and C

Antigen	Source	Clone	ng/test	Fluorochrome	Excitation (nm)	Longpass Dichroic Mirror	Bandpass filter
CD45RO	Biolegend	UCHL1	2500	Brilliant violet 421	405	blank	450/50
CD4	Biolegend	OKT4	150	Brilliant violet 510	405	505LP	525/50
CD45RA	Biolegend	HI100	400	AF488	488	505LP	530/30
CD3	eBioscience	UCHT1	500	PerCP‐Cy5.5	488	685LP	695/40
CD8	Biolegend	HIT8a	2500	AF700	640	685LP	710/50
Viability marker	eBioscience	Cat# 65‐0865	1:1000	eF780	640	755LP	780/60
FoxP3	BD	259D/C7	500	PE	561	blank	582/15
CD3	BD	UCHT1	500	BUV395	355	blank	

**Table 6:** Antibodies used in clinical study (Fig. 2D)

Antigen	Source	Clone	ng/test	Fluorochrome	Excitation (nm)	Longpass Dichroic Mirror	Bandpass filter
CD45RO	Biolegend	UCHL1	600	APC	640		675/12
CD4	Biolegend	OKT4	500	PerCP/Cy5.5	488	670 nm	
CD45RA	Biolegend	HI100	1000	AF488	488		530/15
FoxP3	BD	259D/C7	500	PE	561		585/20

### Quantification of maternal microchimerism

Cord blood lymphocytes were isolated as described above. DNA from 2 to 5 ml of maternal saliva and 5 million cord blood lymphocytes was isolated using the DNEasy Blood and Tissue Kit (Qiagen, Inc., Valencia, CA). A quantitative PCR (qPCR) method detecting mother‐fetus polymorphisms in insertion/deletion regions on different chromosomes that show high natural polymorphisms was applied as published previously 12. Briefly, utilizing In/Del primers (Table 7), corresponding DNA sequences were amplified in both maternal and fetal DNA using a standard PCR reaction and visualized on a 1.5% agrose gel using ethidium bromide. DNA sequences present in maternal DNA but absent in the matched fetal DNA were then further analyzed using qPCR. Dilutions of unrelated DNA samples were used to establish a standard curve for each primer. Utilizing the standard curve, we estimated the concentration of maternal DNA in the DNA equivalent of 1 × 10e6 fetal lymphocytes.

**Table 7:** qPCR primer sequences used for the analysis of microchimerism

Marker		5′‐Primer‐3′
SO‐1a	Forward	GGTACCGGGTCTCCACATGA
	Reverse	GGGAAAGTCACTCACCCAAGG
SO‐2	Forward	GCTTCTCTGGTTGGAGTCACG
	Reverse	GCTTGCTGGCGGACCCT
SO‐3	Forward	CTTTTGCTTTCTGTTTCTTAAGGGC
	Reverse	TCAATCTTTGGGCAGGTTGAA
SO‐4a	Forward^a^	CTGGTGCCCACAGTTACGCT
	Reverse	AAGGATGCGTGACTGCTATGG
SO‐4b	Forward	Same as SO‐4a
	Reverse	AGGATGCGTGACTGCTCCTC
SO‐6	Forward	CAGTCACCCCGTGAAGTCCT
	Reverse	TTTCCCCCATCTGCCTATTG
SO‐7a	Forward	TGGTATTGGCTTTAAAATACTGGG
	Reverse	TGTACCCAAAACTCAGCTGCA
SO‐7b	Forward	GGTATTGGCTTTAAAATACTCAACC
	Reverse	CAGCTGCAACAGTTATCAACGTT
SO‐8a	Forward	CTGGATGCCTCACTGATCCA
	Reverse*	TGGGAAGGATGCATATGATCTG
SO‐8b	Forward	F GCTGGATGCCTCACTGATGTT
	Reverse	Same as SO‐8a
SO‐9a	Forward	GGGCACCCGTGTGAGTTTT
	Reverse	TCAGCTTGTCTGCTTTCTGGAA
SO‐11a	Forward	TAGGATTCAACCCTGGAAGC
	Reverse	CCAGCATGCACCTGACTAACA

^a^ Primers used in more than one sequencing reactions.

### Statistical analysis

Differences in normally distributed populations were statistically analyzed using unpaired Student's *T* testing or analysis of variance (ANOVA). Statistical analyses were accomplished with PRISM (Graphpad, La Jolla, CA).

## Results

### Cord blood from fetuses with placentas implanted over a uterine scar displays increased maternal microchimerism

Trafficking of maternal cells into the fetus is a common phenomenon, as much as 0.1–0.5% cord blood lymphocytes are of maternal origin 9, 13. This trafficking is observed in spite of full uterine integrity, a barrier that currently is considered as shielding the fetus from outside influences. We hypothesized that impairment of uterine integrity, such as scar formation in response to a cesarean section, results in increased trafficking of maternal cells into the fetus when the placenta is in direct contact with the scar tissue. To test this hypothesis, we used a published protocol 12 involving qPCR to quantify the number of maternal cells in cord blood from twenty fetuses born to mothers that had undergone prior cesarean sections. We found that the number of maternal lymphocytes in cord blood of fetuses when the placenta was implanted away from the scar (posterior placenta) was significantly lower than in cord blood from fetuses whose placenta was implanted over the scar (anterior placenta, Fig. 1, *p* = 0.0290). With our quantification method, we detected approximately 1 maternal cell per 1000 fetal cells in fetuses with anterior placentas, an amount roughly 1/5th of that in fetuses with posterior placentas (anterior placenta 0.9998 ± 0.3801, posterior placenta 0.1808 ± 0.0055 maternal cells per 1000 fetal cells).

### Placental interaction with uterine scar determines fetal Treg phenotype

The presence of maternal cells in the fetal environment induces the generation of Tregs with specificity for NIMA. These NIMA‐specific Tregs are thought to be peripheral rather than thymic in origin and dependent on TGF‐β for their generation 9, 14. We observed a higher load of maternal antigen in fetuses whose placenta implanted over a prior uterine scar. The fetus could cope with a higher antigen load (increased maternal microchimerism) by expanding the Treg population or by enhancing functionality of existing Treg. In adults, antigen‐experienced, activated Tregs can be identified by increased expression of FoxP3 and a lack of CD45RA‐expression 15. We hypothesized that placental implantation over the uterine scar would alter the phenotype of the fetal Tregs toward an antigen‐experienced/activated phenotype. To address this hypothesis, we quantified the CD45RA‐ and CD45RO‐expression in the Treg populations of fetuses born to mothers with prior cesarean sections (Fig. 2B and C, Supplemental Fig. S1). When the placenta was implanted away from the scar, cord blood lymphocytes contained T cells and Tregs that were largely CD45RA+ (Fig. 2B). Only fetuses with the placenta implanted over the uterine scar contained a detectable population of CD45RO+ Tregs (Fig. 2C). To confirm this observation, we performed a prospective collection of cord blood samples from otherwise healthy pregnancies with a history of prior cesarean delivery. The cord blood of 10 fetuses with posterior placentas and 10 fetuses with anterior placentas was analyzed for the expression of CD45RO in the FoxP3+ population (Fig. 2D). Of the 10 fetuses whose placentas were implanted posteriorly, one showed CD45RO+ Tregs while the other 9 did not show this phenotype (Fig. 2D). In contrast, the cord blood of all 10 fetuses with anterior placentas contained a distinct CD45RO+ Treg population (Fig. 2D). In our study with 20 participating patients, we found a risk difference of 80% for placental implantation over a prior cesarean section scar resulting in CD45RO+ Tregs in the cord blood (Risk Ratio = 5 [*p* < 0.05 CI:(1.448 to 17.27)]).

### Fetal CD45RO+ Tregs are functionally similar to adult activated Treg

**Figure 3:**
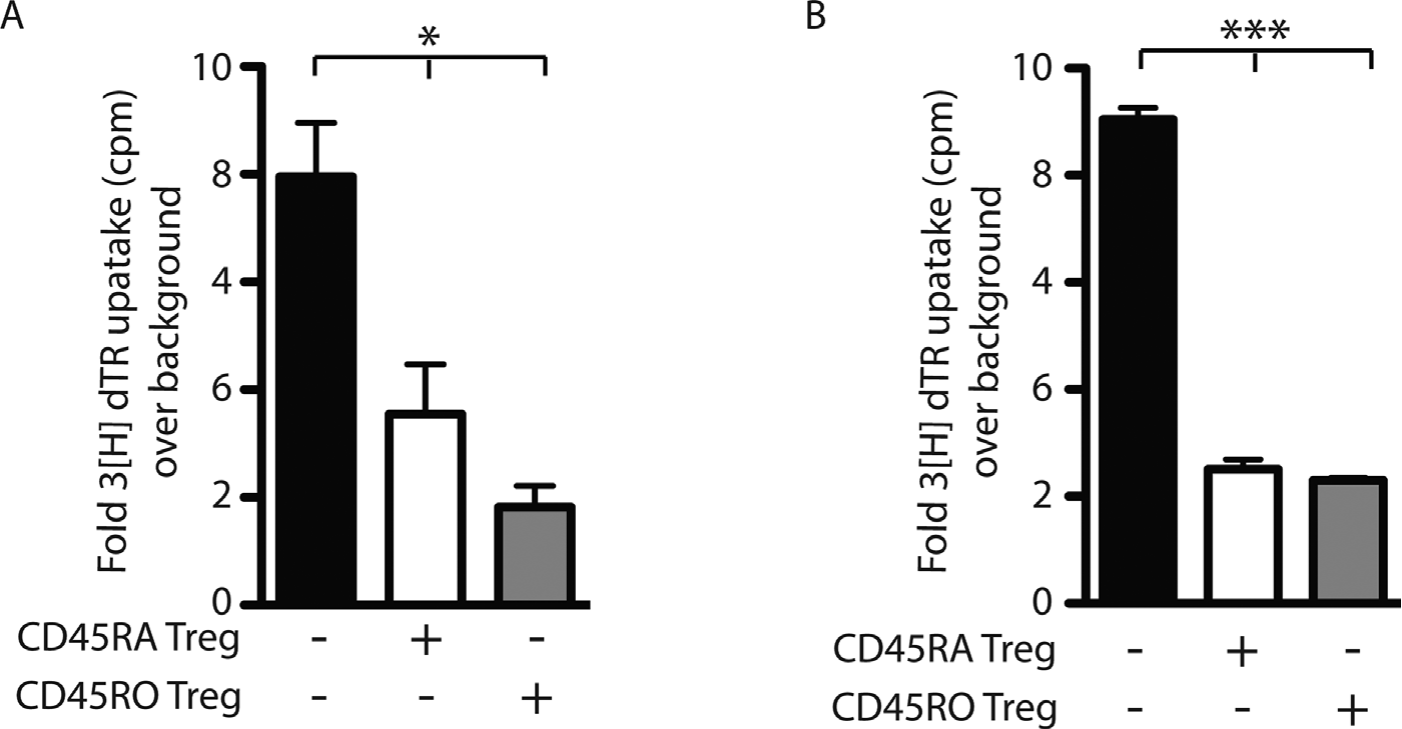
Fetal CD45RO+ Treg are highly suppressive. In vitro suppression assays of cord blood lymphocytes (CBL). (A) Proliferation of total CBL stimulated with ConA with no Treg (black bar), CD45RA+ (gray bar 1:10) or CD45RO+ Treg (white bar 1:10) added. Depicted is a representative result from four independent experiments. Ordinary one‐way ANOVA *p = 0.0135. (B) Proliferation of CD4+ Treg depleted (CD25+ CD127lo) CBL stimulated with irradiated total maternal lymphocytes (1:1) with no fetal Treg (black bar), CD45RA+ (gray bar 1:10) or CD45RO+ Treg (white bar 1:10) added. Depicted is a representative result from three independent experiments (ditto). Ordinary one‐way ANOVA ***p = 0.0001.

In humans, Treg subtypes are identified by surface expression of CD45RA in combination with intranuclear expression of FoxP3: resting Treg (CD45RAhi FoxP3lo), Cytokine Treg (CD45RA‐FoxP3lo) and Activated Treg (CD45RA‐FoxP3hi) 15. In adults, the three subtypes have distinct functional characteristics with Activated Treg displaying the strongest suppression but the lowest proliferation potential 15. CD45RA and CD45RO expression are mutually exclusive and therefore we hypothesized that the FoxP3+CD45RO+ Tregs observed in cord blood lymphocytes from fetuses with anterior placentas represented the CD45RA‐FoxP3hi Activated Treg population described by Miyara et al. 15. Further, in a standard proliferation assay, fetal CD45RA+ Tregs were less suppressive than fetal CD45RO+ Tregs (Fig. 3A).

### Cord blood Treg suppress fetal T cell responses to maternal alloantigens

Similar to the maternal immune system, the fetal immune system is “aware” of maternal antigens, but T cell effector responses are subdued by fetal Tregs in an antigen‐specific manner, as demonstrated in Treg depletion experiments 9. As expected, when CD4+ Treg‐depleted (CD25+CD127lo) cord blood lymphocytes were exposed to irradiated maternal lymphocytes, a subtle proliferative response was observed, reflecting antigenic awareness of the NIMA. Addition of fetal Tregs (either CD45RA+ or CD45RO+) resulted in significant suppression reflecting the antigen‐specific nature of the fetal Treg response to NIMA (Fig. 3B).

## Discussion

The birth of a baby is an important milestone in people's lives; increasingly women choose to undergo cesarean section as a choice informed by concerns other than medical necessity. To date, conversations around elective c‐section largely revolve around concerns for the mother but impacts on the fetuses of future pregnancies have thus far not been included. We found increased trafficking of maternal cells into the fetus and the formation of highly functional memory T reg with specificity for NIMA in those fetuses where the placenta implants over a prior uterine scar. Thus, we have demonstrated that an increasingly common surgical intervention creates conditions that alter the fetal immune repertoire.

Our findings and reports by others suggest that the fetus can be exposed to antigens from maternal circulation across the placent. For example, in utero exposure to malaria (due to placental infection with *Plasmodium*) was found to elicit Treg and functional, tolerogenic responses toward the parasite in the fetus if this exposure occurred early during pregnancy 16, 17, 18, 19. Such exposure can be associated with adverse outcomes in these fetuses such as reduced weight at birth or functional tolerance with an associated higher infection susceptibility and anemia 19, 20. The complexity of in utero antigen exposure and its subsequent effects are reflected in the fact that the cord blood of only a portion of children whose placentae presented with evidence of *Plasmodium* infection in the study by Malhotra et al displayed a tolerogenic response, or in fact any response, toward *Plasmodium* antigens indicating that not all exposures result in fetal immune recognition 19, 21. Further, other maternal disease states such as type 1 diabetes and preeclampsia can also alter the fetal immune system 22, 23. The question raised by our study pertains to mothers infected or possibly even vaccinated during pregnancy who have a uterine scar from either a prior c‐section or other surgical intervention: if the placenta abuts the uterine scar, that situation may chronically expose fetuses to infectious agent—or vaccine antigens, an important topic of future research as such exposure may lead to a fetal Treg response with vaccine antigen specificity, that is likely to persist into childhood, and could therefore contribute to subsequent vaccine failure.

For our microchimerism studies, we used the method described by Lee et al 12. Using our adapted quantification protocol, we detected approximately 1 maternal cell per 1000 fetal cells in fetuses with placentas implanted over the scar and roughly five times more in fetuses with placentas implanted away from the scar (anterior placenta 0.9998 ± 0.3801, posterior placenta 0.1808 ± 0.0055 maternal cells per 1000 fetal cells). Thus, our observed values are within the reported range of maternal‐fetal microchimerism although those previous studies did not address history of cesarean section 9, 13.

CD45RA and CD45RO expression on T cells are mutually exclusive and therefore we hypothesized that the FoxP3+CD45RO+ Tregs observed in cord blood lymphocytes from fetuses with anterior placentas represented the CD45RA‐FoxP3hi Activated Treg population described by Miyara et al. 15. We found that in a standard proliferation assay, fetal CD45RA+ Tregs were less suppressive than fetal CD45RO+ Tregs (Fig. 2A). These results indicate that cord blood Treg phenotype and functional characteristics are similar to those from adults and that the CD45RO+ Treg population observed in anterior placenta fetuses represents an activated type of Treg as classified by Miyara et al. 15.

Taken together, our study adds a novel aspect to an important field of research, the mechanism of antigen transfer during antigenic exposure of infants in utero: uterine integrity. While the impact of in utero antigenic exposure is only partially understood, the mechanisms are even less clear. We found that uterine scars from prior c‐sections allow for increased maternal—to fetal cell trafficking; in how far it also allows for increased trafficking of other antigens is subject of current studies. Given that, at least in some circumstances, in utero fetal antigen exposure can elicit inappropriate tolerance that lasts for years, further studies are urgently needed to understand the implication of these observations for maternal vaccination during pregnancy.

## Ethics Statement

Human subjects were recruited for participation after IRB approval (University of California, Los Angeles, Office of Human Research Protection Program, Medical IRB Committee‐1 #11‐003962). Each subject provided written informed consent prior to enrollment.

## Supporting information

Additional supporting information may be found in the online version of this article at the publisher's web‐site.

**Figure S1**. Gating strategy and CD45RA/RO distribution on adult T cells and Treg.
